# Epidemiology of *Plasmodium* spp. Detection Among Acute Febrile Illness Patients in Two Regions of Nigeria

**DOI:** 10.1093/cid/ciaf468

**Published:** 2025-11-20

**Authors:** Claire A Quiner, Adamu Zigwai Ephraim, Vivian Kwaghe, Cyril Erameh, Jay Samuels, Lauren P Courtney, Jean H Kim, Osahogie Isaac Edeawe, Nankpah Vongdip, Onyia Justus Ejike, Ikponmwosa Odia, Kat Asman, Philippe Chebu, Jacqueline Agbukor, Oladimeji Damilare Matthew, Victoria Orok, Femi Owolagba, Blessed Okhiria, Ephraim Ogbaini-Emovon, Walter Mary Odion, Blessing Amierhobhiye Obagho, Richard Fayomade, Joella Adams, Emmanuel A Oga

**Affiliations:** Solutions, RTI International, Durham, North Carolina, USA; Solutions, RTI International, Durham, North Carolina, USA; Internal Medicine, University of Abuja Teaching Hospital, Gwagwalada, Federal Capital Territory, Nigeria; Institute of Viral and Emergent Pathogens Control and Research, Irrua Specialist Teaching Hospital, Edo, Nigeria; Laboratory Services, APIN Public Health Initiatives, Federal Capital Territory, Nigeria; Solutions, RTI International, Durham, North Carolina, USA; Solutions, RTI International, Durham, North Carolina, USA; Institute of Viral and Emergent Pathogens Control and Research, Irrua Specialist Teaching Hospital, Edo, Nigeria; Internal Medicine, University of Abuja Teaching Hospital, Gwagwalada, Federal Capital Territory, Nigeria; Internal Medicine, University of Abuja Teaching Hospital, Gwagwalada, Federal Capital Territory, Nigeria; Institute of Viral and Emergent Pathogens Control and Research, Irrua Specialist Teaching Hospital, Edo, Nigeria; Solutions, RTI International, Durham, North Carolina, USA; Laboratory Services, APIN Public Health Initiatives, Federal Capital Territory, Nigeria; Institute of Viral and Emergent Pathogens Control and Research, Irrua Specialist Teaching Hospital, Edo, Nigeria; Internal Medicine, University of Abuja Teaching Hospital, Gwagwalada, Federal Capital Territory, Nigeria; Internal Medicine, University of Abuja Teaching Hospital, Gwagwalada, Federal Capital Territory, Nigeria; Laboratory Services, APIN Public Health Initiatives, Federal Capital Territory, Nigeria; Internal Medicine, University of Abuja Teaching Hospital, Gwagwalada, Federal Capital Territory, Nigeria; Institute of Viral and Emergent Pathogens Control and Research, Irrua Specialist Teaching Hospital, Edo, Nigeria; Institute of Viral and Emergent Pathogens Control and Research, Irrua Specialist Teaching Hospital, Edo, Nigeria; Institute of Viral and Emergent Pathogens Control and Research, Irrua Specialist Teaching Hospital, Edo, Nigeria; Laboratory Services, APIN Public Health Initiatives, Federal Capital Territory, Nigeria; Solutions, RTI International, Durham, North Carolina, USA; Solutions, RTI International, Durham, North Carolina, USA; ClineEpi Partners, Columbia, Maryland, USA

## Abstract

**Background:**

Malaria is a common cause of acute febrile illness (AFI) in Nigeria.

**Methods:**

Patients presenting with AFI at two tertiary hospitals were enrolled from August 2023 to September 2024. Specimens were screened for 25 pathogens using a multi-pathogen screening assay. Demographic, seasonal, clinical, and environmental associations with *Plasmodium* spp. detection via the assay are described and analyzed. An exploratory analysis of climatic predictors for percent positivity was conducted. Other pathogens detected, alongside *Plasmodium* spp., are reported.

**Results:**

*Plasmodium* spp. was detected in 293 (24.4%) enrollees. Larger household size (crude odds ratio [COR] = 3.70, 95% confidence interval [CI] = 1.12–12.28), being a healthcare worker (COR = 3.39, CI = 1.22–9.41), children aged 11–14 (COR = 1.86, CI = 1.15–3.02), enrollment from FCT (COR = 1.30, CI = 1.00–1.69), and start of the dry season were associated with an increased risk for *Plasmodium* spp. detection. Coinfections were detected in 37.2% (n = 109) of the enrollees with *Plasmodium* spp. detected. Among these, nine biodefense pathogens, per the National Institute of Allergy and Infectious Diseases’ list, were detected in 105 enrollees. No statistical significance among climatic predictors was found; however, a systematic directionality in minimum temperature effects across lag periods was observed. The magnitude of association varied by site with percent positivity in Edo State being more influenced by minimum temperature.

**Conclusions:**

These findings suggest the need for updated diagnostic options in healthcare facilities and expanded surveillance for emerging health threats. An expanded comparative analysis of differential climatic predictors for ecologically distinct regions may reveal insights relevant to prevention efforts.

Malaria, caused by one of four *Plasmodium* single-celled parasitic protozoa (spp.) parasites, *P. falciparum*, *P. vivax*, *P. malariae*, *P. ovale,* and *P. knowlesi* remains a formidable challenge for public health in Nigeria. In 2021, Nigeria reported 68 million cases, accounting for roughly 27% of malaria cases globally [1]. Malaria accounts for 60% of outpatient hospital visits in Nigeria [2], placing a significant strain on the resource-limited health system. This burden of disease underlines the urgent need for sustained and effective interventions, informed by the current epidemiology and risk factors driving malaria incidence in affected populations.

*Plasmodium* spp. are transmitted to humans via a mosquito vector, *Anopheles gambiae*, as climates throughout Nigeria are favorable to this species' survival and propagation [3]. Temperature and precipitation are critical predictors in the occurrence, timing, and intensity of malaria incidence, as the *Anopheles* vector life cycle is sensitive to even minor changes in these factors. Malaria cases tend to peak during the rainy season in each micro-climate throughout Nigeria, due to the abundance of standing water, such as puddles, open containers, and poorly drained areas, which facilitate mosquito propagation [3, 4].

Socioeconomic and demographic factors also critically influence malaria incidence. Populations with lower socioeconomic status often have restricted access to preventive measures that protect against *Plasmodium* spp. transmission and treatment facilities that provide adequate care for infected individuals [5, 6]. Demographic characteristics, particularly age and sex, are known to drive differential malaria incidence among specific groups, including young children, pregnant women, and young men. Young children are at higher risk due to their underdeveloped immune systems, which limits their ability to control *Plasmodium* spp. infections effectively [7, 8]. In certain regions, young men have been reported to have higher exposure to mosquito bites. This has been attributed to occupational and behavioral factors, which can increase their risk of exposure, as compared with women in the same age groups [9, 10].

These demographic characteristics, particularly age and sex, suggest that distinct demographic groups each experience unique epidemiological dynamics driven by their differential risk factors. Exploring these interconnected demographic and climatic predictors, as they vary by region, is important for building a comprehensive epidemiological understanding of malaria within the region, which can contribute to the design of targeted malaria interventions and allocating resources effectively to reduce the disease burden in the most susceptible populations.

In Nigeria, the Federal Capital Territory (FCT) and Edo State both benefit from malaria interventions primarily aimed at reducing the disease burden amongst vulnerable groups, particularly children. For instance, the National Malaria Strategic Plan (2014–2020) implemented by the National Malaria Elimination Programme, Nigeria's government agency for malaria control, provided chemoprevention therapy to over 12 million children under age 5 in 101 of the country's 775 (13.0%) local government areas (LGAs) [11].

In addition to the Nigerian government's role, non-governmental organizations and international organizations have played a critical role in supplementing the government's capacity to tackle the disease burden effectively by providing financial support, materials, learning resources, and expertise. Non-profit organizations, like Malaria Consortium, through the Seasonal Malaria Chemoprevention (SMC) Impact Project bolster government-led interventions by administering monthly antimalarial medication—with almost 10 million children under 5 years of age reached in 2020 alone [12]. Other initiatives include the distribution of insecticide-treated nets through campaigns and community-based programs, supported by international organizations committed to reducing malaria prevalence, especially among children [13]. One of these initiatives is the President's Malaria Initiative implemented by the U.S. government, which procured more than 60 million insecticide-treated nets as part of its efforts toward malaria control in Nigeria [14]. Despite these successes, the burden of malaria remains substantial across Nigeria, responsible for 26% of global malaria cases recorded in 2023 [15]. Accordingly, the epidemiology of malaria should be continuously monitored and tracked to inform the design or use of interventions aimed at reducing *Plasmodium* spp. transmission.

The Surveillance of Acute Febrile Illnesses in Nigeria (SAFIAN) study utilized a molecular-based, multi-pathogen detection platform, Thermo Fisher TaqMan™ Array Card (TAC) [16] to assess acute febrile illness (AFI) patients presenting at two hospitals in Nigeria: Gwagwalada, FCT, representing a savannah climate with a mostly suburban population, and Irrua, Edo State, representing a rainforest region in the south of Nigeria with a mostly rural populations. Patient specimens were screened for *Plasmodium* spp., as well as 25 other molecular pathogenic targets using TACs.

This analysis aims to provide a descriptive epidemiological and comparative analysis of the incidence of *Plasmodium* spp. detection in these two regions of Nigeria.

## METHODS

A comprehensive description of the methods, procedures, and details of the SAFIAN study can be found in detail in Courtney et al (2025). They are described in brief herein.

### Enrollment

The SAFIAN study was conducted in two hospitals in Nigeria: Irrua Specialist Teaching Hospital (ISTH) in Edo State, and University of Abuja Teaching Hospital (UATH) in the FCT. Patient enrollment spanned August 2023 to September 2024, with participants recruited in each of the 12 months of the year. Patients at each hospital were identified by hospital staff, and local SAFIAN study staff screened and enrolled them into SAFIAN if they met the criteria for AFI, had a measured temperature of ≥37.5°C or a history of fever within the preceding 10 days and were aged ≥5 years. For medical treatment, patients were tested, diagnosed, and treated by hospital physicians, per hospital protocols. After patient eligibility was confirmed and consent/assent was given, participants were enrolled and given a ₦4000 incentive.

### Data and Specimen Collection

Blood and serum samples were collected by a phlebotomist. Clinical, socio-demographic, and other data were collected using standardized Research Electronic Data Capture (REDCap) electronic data forms.

### Specimen Analysis

All blood samples collected for the SAFIAN study (n = 1200) were analyzed for *Plasmodium* spp., as well as 24 other pathogens using TAC and supported by extensive quality control protocols (Courtney et al 2025). TaqMan™ Array Card is a molecular based, research-use only, screening assay with targets customized to the study. Assays for each pathogenic target were designed and tested for specificity and sensitivity by Thermo Fisher Scientific. These assays are proprietary. The assay for *Plasmodium* spp. did not differentiate among the *Plasmodium* species.

### Statistical Analysis

Data integration, analysis and map generation were conducted using R Studio 2023.06.0 (Boston, MA, USA). Analyses to examine univariate associations between *Plasmodium* spp. detection and various variables were calculated using odds ratio by median-unbiased estimate and mid-p exact confidence intervals [17]. Chi-squared tests were used to determine statistical significance. The SAFIAN study was designed to enroll more adults than children (60% adults, 40% children). Accordingly, demographics impacted by this age designation are analyzed by age classification, to avoid bias in analyses. Age-dependent demographics such as age and educational status were analyzed separately for adults and children. Environmental data were accessed through the Google Earth Engine Platform. CHIRPS Daily: Climate Hazards Center InfraRed Precipitation with Station Data (Version 2.0 Final) was used for precipitation estimates and the temperature data were provided by the National Oceanic and Atmospheric Administration Physical Sciences Laboratory, Boulder, Colorado, USA.

A generalized linear mixed model with a logit link and binomial distribution (PROC GLIMMIX, SAS) was used to assess the relationship between weather variables and weekly percent positivity for *Plasmodium* spp. detection, at each site. Given the timing and dynamics of the larval development of mosquitoes, we tested associations with time lagged weather-related variables (precipitation and minimum and maximum temperature) from 0 to 6 weeks, preceding enrollment into the SAFIAN study (the time when *Plasmodium* spp. was detected in that enrollee). Mean daily temperatures can influence the rate of parasite development [18] including the length of the gonotrophic cycle, fecundity, biting rate, longevity, and development of immature mosquitoes [19]. Even small changes in mean or diurnal temperature, relative humidity, and precipitation can impact the life cycle of vectors and their parasites. These changes, in part, eventuate in the timing and intensity of parasitic transmission to humans [20]. Accordingly, using a time lagged approach, weather variables from 0 to 6 weeks prior to the time of *Plasmodium* spp. detection, allows for the influence of weather predictions, at different points in the vector and parasitic development cycle, to predict *Plasmodium* spp. incidence in humans. Moreover, weather conditions may predict incidence differently in different ecosystems, accordingly, this analysis was conducted for each site of enrollment separately. The enrollment site in Edo state is proximal to Nigeria's Atlantic coastline, and the other, in Abuja, is more inland.

## RESULTS

Of the 1200 SAFIAN participants enrolled over the 12-month period, *Plasmodium* spp. was detected in 293 (24.4%). Prevalence of *Plasmodium* spp. varied by site of enrollment, month, demographic, and LGA of residence.

**Figure 1.**
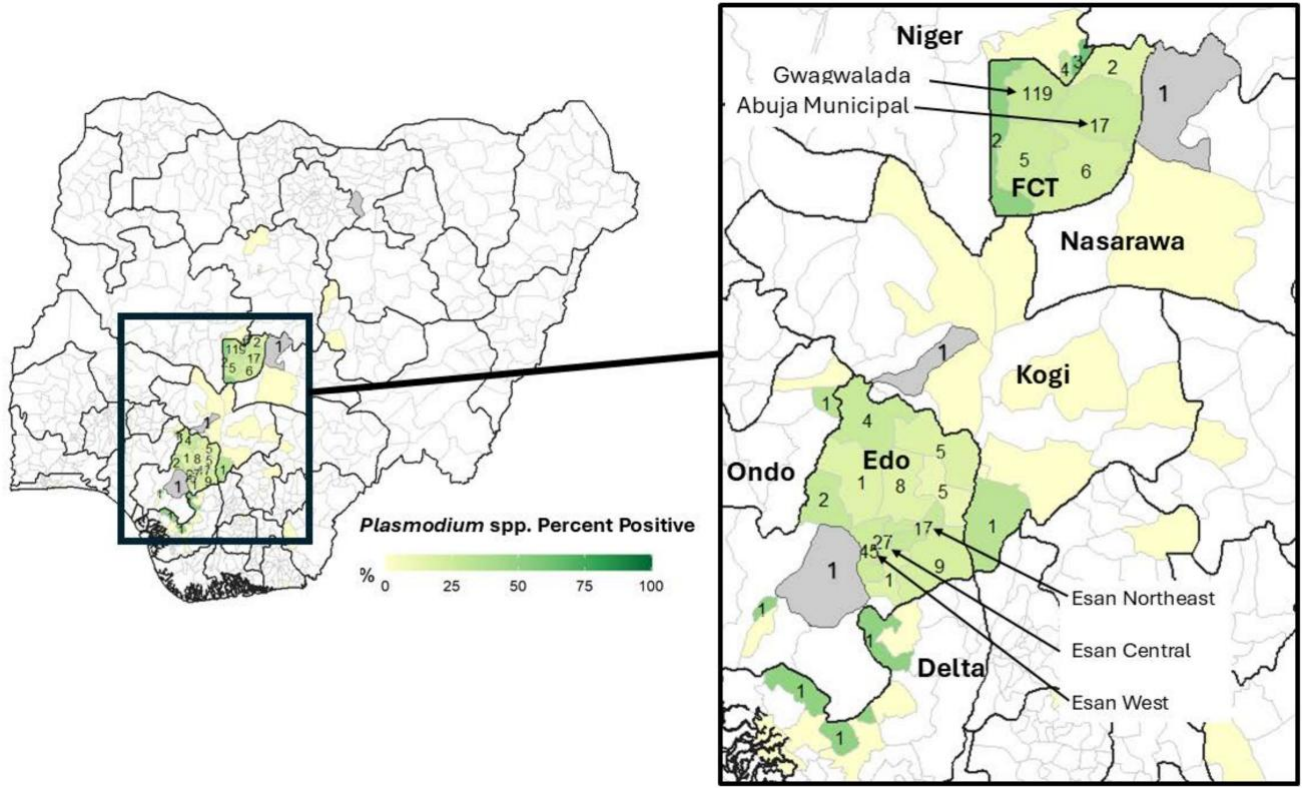
Percentage of *Plasmodium* spp. positive enrollees by LGA. Numbers indicate the count of enrollees who tested positive in each LGA. Grey-shaded LGAs represent areas where all enrollees tested positive for *Plasmodium* spp. (100% positivity). Black lines are state boundaries, grey lines are LGAs. Abbreviations: FCT,Federal Capital Territory; LGA, local government area.

*Plasmodium* spp. detection was not uniform across the LGAs of residence (Figure 1). However, among the LGAs with the highest numbers of enrollees, Gwagwalada (n = 434), Esan West (n = 185), and Esan Central (n = 108), prevalence was approximately the same in each (27.4%, 24.3%, 25.0%, respectively) over the 2023–2024 study period. The colorimetric scale reflects the prevalence of *Plasmodium* spp. detection among SAFIAN enrollees from each LGA. The number reflects the count of *Plasmodium* spp.-positive enrollees in each LGA. Pale yellow LGAs without numbers had SAFIAN enrollees, but no *Plasmodium* spp. cases detected. White LGAs indicate that no participants were enrolled from them. Gray LGAs had 1 enrollee and that enrollee tested positive for *Plasmodium* spp. (100% prevalence). Bolded names are states. Other names are LGAs.

Table 1 shows the crude OR of *Plasmodium* spp. detection by age, educational level attained, and occupation. Age was significantly associated with *Plasmodium* spp. detection among the children. Children aged 11–14 had 1.86 times the risk of younger children. Among adults, education, marital status, and age group were not associated with the risk; however, occupation was. Healthcare workers had 3.36 times the risk of *Plasmodium* spp. detection compared with farmers, followed by students (COR = 2.68).

**Table 1.** Age-Dependent Demographics of SAFIAN Enrollees with *Plasmodium* spp. Detection

Demographic	Status	*Plasmodium* spp. Detected n (%)	Crude Odds Ratio	95% Confidence Interval	*P* Value
**Children**					
Age	5–10 y old	46 (25.3%)	(REF)	…	.062
	11–14	51 (35.2%)	1.86	(1.15, 3.02)	…
	15–17 y old	48 (33.1%)	1.61	(.99, 2.62)	…
Education	No formal education/below primary	26 (22.2%)	(REF)	…	…
	Primary	55 (39.0%)	2.23	(1.29–3.92)	.005
	Below secondary	3 (33.3%)	1.78	(.34–7.5)	.429
	Secondary	52 (34.9%)	1.87	(1.08–3.28)	.030
	Tertiary	7 (26.9%)	1.30	(.46–3.36)	.612
	Other/more than one picked	2 (25.0%)	1.21	(.15–5.88)	1.00
Occupation	Student	137 (32.2%)	(REF)	…	…
	Any other occupation	8 (32.0%)	0.9992	(.39–2.32)	1.0
**Adults**					
Age	18–49 y old	112 (75.7%)	(REF)	…	…
	50+ years old	36 (24.3%)	0.75	(.49, 1.13)	.189
Education	No formal education/below primary	8 (11.4%)	(REF)	…	…
	Primary	6 (16.7%)	1.55	(.49, 4.87)	.547
	Below secondary	5 (13.5%)	1.21	(.37, 4.00)	.763
	Secondary	59 (23.7%)	2.41	(1.09, 5.31)	.030
	Tertiary	63 (19.7%)	1.90	(.87, 4.17)	.124
	Other/more than one picked	7 (18.4%)	1.75	(.58, 5.27)	.385
Occupation	Farmer	8 (13.3%)	(REF)	…	…
	Trader	22 (15.8%)	1.22	(.51, 2.93)	.829
	Artisan	12 (22.6%)	1.90	(.71, 5.09)	.224
	Student	26 (29.2%)	2.68	(1.12, 6.42)	.029
	Civil servant	26 (20.0%)	1.62	(.69, 3.84)	.313
	Housewife/unemployed/retired	23 (18.3%)	1.45	(.61, 3.47)	.528
	Skilled/Unskilled manual/miner/transporter	5 (9.8%)	0.71	(.22, 2.31)	.768
	Healthcare workers	12 (34.2%)	3.39	(1.22, 9.41)	.020
	Other occupations	14 (20.9%)	1.72	(.66, 4.44)	.349
Marital status	Married	87 (18.1%)	0.75	(.52, 1.09)	.151
	Not married	61 (22.7%)	(REF)	…	…

Abbreviation: SAFIAN, Surveillance of Acute Febrile Illness Aetiology in Nigeria.

Among non-age dependent demographic and behavioral risk factors, site of enrollment was significantly associated with *Plasmodium* spp., with enrollees from FCT having 1.30 times the risk for *Plasmodium* spp. as compared with those from Edo State (Table 2). Having a smaller household size reduced the odds of *Plasmodium* spp., with enrollees from small- (1–5 people) and medium-sized (6–10 people) households having 0.27 and 0.42 times the risk of those living in a large (11+ people) household. *Plasmodium* spp. detection did not differ statistically by participants' sex, bed net usage in the last week, time spent in the forest in the last 2 weeks, or insect bites in the last 2 weeks. Chronic diseases including cancer and diabetes were also not statistically different among participants with or without *Plasmodium* spp. detection (data not shown).

**Table 2.** Demographics and Behavioral Risk Factors Among SAFIAN Enrollees With *Plasmodium* spp. Detection

Demographic	Characteristic	*Plasmodium* spp. Detected n (%)	Crude Odds Ratio	95% Confidence Interval	*P* Value
Sex	Male	157 (22.2%)	1.27	(.98, 1.66)	.979
	Female	136 (26.7%)	(REF)	…	…
Enrollment site	FCT	161 (26.8%)	1.30	(1.00, 1.69)	.059
	Edo State	132 (22.0%)	(REF)	…	…
Bed net usage in the past week	0 d	188 (23.3%)	(REF)	…	…
	1–6 d	55 (27.2%)	1.23	(.87, 1.75)	.270
	7 d	50 (%)	1.16	(.81, 1.66)	.451
Time spent in forest in the past 2 wks	0 d	240 (25.2%)	(REF)	…	…
	1–7 d	53 (21.3%)	0.80	(.57, 1.12)	.214
Insect bite in the past 2 wks	Yes	163 (%)	1.12	(.84–1.47)	.475
	No	106 (%)	(REF)	(.82, 2.21)	…
	Don’t Know	24 (%)	0.74	(.44–1.21)	.280
Household size	1–5 people	165 (21.2%)	(REF)	…	…
	6–10 people	122 (29.7%)	1.57	(1.19–2.06)	.001
	11+ people	6 (50.0%)	3.70	(1.12–12.28)	.027

Abbreviation: SAFIAN, Surveillance of Acute Febrile Illness Aetiology in Nigeria.

Table 3 shows the clinical profile of participants with *Plasmodium* spp. detection. Participants with jaundice had 3.6 times the odds of a *Plasmodium* spp. detection and those with vomiting or nausea had 1.4 times the odds. Those with diarrhea had 0.54 times the odds of *Plasmodium* spp. detection. This analysis was a part of a larger surveillance study that screened for 25 pathogens in 1200 patient specimens, using a multi-pathogen TAC assay [16]. The other pathogens detected, among the *Plasmodium* spp.-positive patients, are shown in Table 4. Of the 293 SAFIAN enrollees where *Plasmodium* spp. was detected, 109 of them (37.2%) had a coinfection with one or more other pathogens. The most common of these coinfections was with *Ricketssia* spp. (25.3%), followed by Lassa fever virus (5.5%). In five of these coinfected patients, three or more different pathogens were detected.

**Table 3.** Symptomatic Characteristics Among SAFIAN Enrollees With *Plasmodium* spp. Detection

Symptom	*Plasmodium* spp. Detected n (%)	Odds Ratio*	95% Confidence Interval	*P* Value
Cough	49 (22.9%)	0.90	(.64, 1.28)	.568
Runny nose	24 (23.5%)	0.95	(.59, 1.53)	.827
Diarrhea	22 (15.7%)	0.54	(.34, .87)	.012
Vomiting/nausea	115 (28.8%)	1.41	(1.07, 1.85)	.014
Stomach pain	71 (25.1%)	1.05	(.77, 1.43)	.762
Rash	4 (18.2%)	0.68	(.23, 2.04)	.495
Headache	204 (25.4%)	1.18	(.89, 1.57)	.257
Joint pain	33 (21.9%)	0.85	(.56, 1.28)	.434
Muscle pain^a^	41 (22.1%)	0.86	(.59, 1.25)	.434
Unusual bleeding	11 (23.4%)	0.94	(.47, 1.88)	.871
Jaundice^a^	9 (52.9%)	3.57	(1.37, 9.35)	.009

Abbreviations: SAFIAN, Surveillance of Acute Febrile illness Aetiology in Nigeria; OR, Odds Ratio.

^a^OR and *P* value based on only those respondents picking yes/no.

^*^“No” was used as the reference groups for each of these statistical tests.

**Table 4.** Coinfections Among Patients With *Plasmodium* spp. Detected via TAC, by Pathogen

TAC Molecular Detections	Percentage of *Plasmodium* spp.-Infected Enrollees With Coinfection
*Plasmodium* spp. (PLAS) only	62.5% (n = 183)
*Rickettsia sp*. (RICK) and PLAS	25.3% (n = 74)
Lassa Fever Virus (LASV)* and PLAS	5.8% (n = 17)
*Neisseria meningitidis* and PLAS	1.0% (n = 3)
PLAS, RICK, and LASV	0.68% (n = 2)
Congo-Crimean hemorrhagic virus and PLAS	0.68% (n = 2)
Chikungunya virus and PLAS	0.68% (n = 2)
*Brucella* spp. (BRUC) and PLAS	0.34% (n = 1)
BRUC, RICK, and PLAS	0.34% (n = 1)
Hepatitis E and PLAS	0.34% (n = 1)
Monkey pox virus and PLAS	0.34% (n = 1)
O'nyong'nyong virus, LASV, and PLAS	0.34% (n = 1)
Dengue virus and PLAS	0.34% (n = 1)
RICK, pan-*Salmonella,* and PLAS	0.34% (n = 1)
*Yersinia pestis* and PLAS	0.34% (n = 1)
Zika virus and PLAS	0.34% (n = 1)

Abbreviations: TAC, TaqMan Array Card; PCR, polymerase chain reaction; SAFIAN, Surveillance of Acute Febrile Infectious Aetiologies in Nigeria.

^*^The LASV results reported here are those detected by TAC, by a hospital diagnostic test and those detected via a follow-up singlet polymerase chain reaction (PCR) test performed by SAFIAN staff [16].

**Figure 2.**
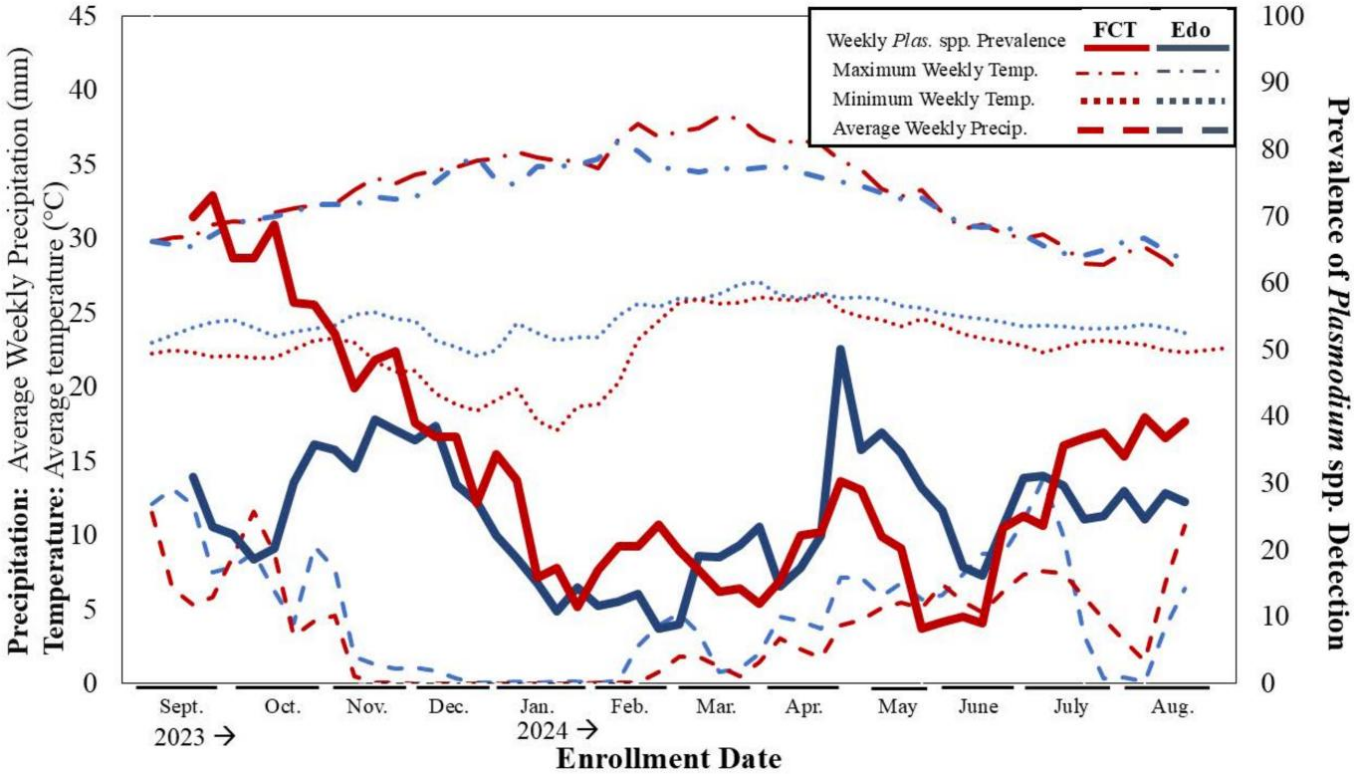
Comparison of prevalence of *Plasmodium* spp. detection to weekly precipitation and temperature averages from each region of enrollment, FCT (red) and Edo state (blue). Abbreviation: FCT, Federal Capital Territory.

Weekly averages of minimum and maximum temperature and precipitation across the catchment regions for enrollment were plotted and compared with weekly *Plasmodium* spp. prevalence rates (Figure 2). Overall, a higher prevalence was observed among participants enrolled in FCT compared to those from Edo State (Table 3). Greater variability in prevalence during the enrollment period was also observed in FCT, as compared with weekly prevalence from Edo State. Similarly, greater temperature variability (in weekly minimum and maximum values) was recorded in FCT than in Edo State. Peak *Plasmodium* spp. prevalence was observed from September to October for FCT and in April to May for Edo State.

Moving weekly averages of precipitation, minimum and maximum temperatures are displayed for FCT and Edo State (left axis). The weekly prevalence of *Plasmodium* spp. detections among SAFIAN enrollees are displayed as solid lines with a moving average transformation (right axis).

Based on these observations, seasonality was further assessed. A statistically significant difference among the four seasonal periods is shown in Table 5. Overall, the highest risk was observed during the start of the dry season (COR = 4.49), and the lowest during the end of the dry season. The Harmattan period, a two-month dry windy period between the two shorter dry seasons, shows a decrease in risk from the dry season; however, the magnitude of the relationship varied across the two sites individually (FCT: COR = 2.12, Edo State: COR = 3.99). See Supplemental Materials for additional data.

**Table 5.** Seasonal Prevalence of *Plasmodium* spp. Detection

Seasonal Period	*Plasmodium* spp. Prevalence N (%)	Crude Odds Ratio	95% Confidence Interval	*P* Value
**Overall**				
Dry Season Start (15 Oct–14 Nov)	32 (40.0%)	4.49	(2.56–7.89	<.001
Harmattan Period (15 Nov–14 Jan)	52 (28.5%)	2.70	(1.70–4.33)	<.001
Dry Season End (15 Jan–15 Mar)	39 (12.9%)	(REF)	…	…
Wet Season (16 Mar–14 Oct)	170 (26.8%)	2.47	(1.70–3.67)	<.001
**FCT**				
Dry Season Start (15 Oct–14 Nov)	19 (48.7%)	4.87	(2.29–10.46)	<.001
Harmattan Period (15 Nov–14 Jan)	25 (29.1%)	2.12	(1.13–3.97)	<.001
Dry Season End (15 Jan–15 Mar)	27 (16.2%)	(REF)	…	…
Wet Season (16 Mar–14 Oct)	90 (29.2%)	2.13	(1.33–3.50)	<.001
**Edo State**				
Dry Season Start (15 Oct–14 Nov)	13 (31.7%)	4.73	(1.94–11.75)	<.001
Harmattan Period (15 Nov–14 Jan)	27 (28.1%)	3.99	(1.93–8.70)	<.001
Dry Season End (15 Jan–15 Mar)	12 (8.82%)	(REF)	…	…
Wet Season (16 Mar–14 Oct)	80 (24.5%)	3.31	(1.79–6.62)	<.001

Abbreviation: FCT, Federal Capital Territory.

To interrogate this relationship further, a generalized linear mixed model with a logit link and binomial distribution (PROC GLIMMIX, SAS) was used to account for correlation and non-normal distribution weekly proportion of SAFIAN enrollees who were positive for *Plasmodium* spp. We evaluated if weather variables (maximum weekly temperature, minimum weekly temperature, and average weekly precipitation) were associated with the percentage of patients enrolled with *Plasmodium* spp. detected for each site individually and for all observations with site included as a predictor. Associations were tested time lagged weather-related variables from 0 to 6 weeks preceding enrollment. Within this exploratory analysis, none of the climate-related variables (at enrollment or lagged) had a statistically significant association with the percent of *Plasmodium* spp. detected at enrollment week, within the model; however, some variables are approaching significance (Supplementary Table 1), and there was a systematic directionality in the effect of minimum temperature, across the lagged times. There was a peak in the magnitude of the effect of the odds of testing positive at lag time of 4 weeks. Also, the association varied by site, with percent positivity in Edo state being more influenced by minimum temperature, as compared with the ability of minimum temperature to predict positivity in FCT.

## DISCUSSION

Among minors, the highest detection rates of *Plasmodium* spp. were observed in the 11- to 15-year age group. This finding may reflect the impact of targeted interventions like the Malaria Consortium's SMC program, which primarily focuses on children under the age of five [12]. As younger children receive prophylactic treatments, children are likely to be older when they experience their primary *Plasmodium* spp. infection, leading to a shift in the burden of disease to the older age group. This same epidemiological shift has been found in other studies where there have been interventions focused on younger children [21, 22]. Additionally, in many households, younger children are often prioritized to sleep under insecticide-treated nets, leaving older children unprotected and more exposed to mosquito bites [23].

A significant association was found between larger household sizes and increased risk of *Plasmodium* spp. detection. This finding is consistent with previous studies, which found that higher density households are associated with increased risk of malaria [24, 25]. Larger households often face overcrowded living conditions, which can limit the ability to provide every household member with mosquito nets or adequate protective measures [26]. In addition, some mosquito species—particularly *Anopheles* vectors—have been shown to be attracted to houses with higher human occupancy [27], thereby increasing the risk of vector contact in higher density households. No significant associations were identified between *Plasmodium* spp. detection and bed net usage, sex, education, marital status, time spent in forested areas, or recent insect bites. The lack of association with bed net usage might be attributed to factors such as inconsistent use, improper maintenance, and the development of insecticide resistance among mosquito populations. Occupation was associated with risk, representing healthcare workers as the highest risk for *Plasmodium* spp. detection.

Jaundice and gastrointestinal symptoms were significantly associated with *Plasmodium* spp. detection. Recent studies show that jaundice appears as a significant symptom across various age groups in Nigeria, influenced by yellow fever, hepatitis viruses, and other diseases [28, 29] and jaundice is not a symptom commonly associated with non-severe malaria. Accordingly, jaundice may not be an accurate indicator of *Plasmodium* spp. infection. There were too few enrolled participants who had this symptom (n = 17) for further interrogation of this finding.

This analysis of seasonal variability showed that the peak and lowest prevalence of *Plasmodium* spp. detection varied slightly by site. In FCT, the peak was in September, at the start of the official dry season, however, before the precipitation and temperature had begun to decrease for a sustained period of time, that year. There was a consistent decline in *Plasmodium* spp. detection from FCT enrollees from October to January, consistent with a decrease in rainfall, across that region. In Edo state, rates were steadier, as compared with FCT rates, throughout the course of the study, however, there was a decline, starting in December, shortly after a decline in precipitation. This trend aligns with established knowledge that increased rainfall creates breeding grounds for *Anopheles* mosquitoes and leads to an increase in malaria incidence [30]. Notably, FCT, which had a larger number of *Plasmodium* spp. detections overall, but where prevalence varied significantly throughout the year, exhibited more temperature fluctuations throughout the year. This compared to Edo State, proximal to Nigeria's Atlantic coastline, had a more stable temperature profile and a more stable *Plasmodium* spp. prevalence. This highlights the importance of examining actual weather measurements, rather than seasons defined by dates, and various in patterns of the same disease, within a single country. Despite the insignificant results of the climate variable modeling, a larger sample size may provide insight into climatic predictors and relevant time lags for distinct locations in Nigeria.

Coinfections were common amongst this AFI population. *Plasmodium* spp. was frequently detected alongside other pathogens of global public health importance [31], including nine pathogens on NIAID's biodefense pathogen list: *Ricketssia* spp., Lassa Fever Virus, Congo-Crimean hemorrhagic virus, *Brucella* spp., monkeypox virus, O'nyong'nyong virus, pan-*Salmonella*, Zika virus, and *Yersinia pestis* [32]. These results, if confirmed, could have significant medical practice, in both treatment and diagnostics, and biosafety ramifications for the region.

There are several other factors, outside the scope of this study, which may drive a change in the epidemiology of malaria. For example, Nigeria's urban areas are experiencing significant population growth, often accompanied by inadequate housing, poor drainage infrastructure, and limited sanitation facilities—conditions that are conducive for mosquito breeding [33].

Additionally, the high density of populations and limited access to healthcare in low-resource urban communities facilitate the survival and propagation of mosquito vectors [34]. These factors help strengthen the impact of urban malaria as a significant public health concern, necessitating tailored control strategies that address the unique challenges of urban settings.

Another variable in flux is new vector competence for *Plasmodium* spp. *Anopheles stephensi,* an invasive species in Africa that has been documented in multiple African countries, including Ethiopia (2016) [35], Sudan (2016), Somalia (2019) [36], Kenya (2022) [37], Ghana (2022) [38], and in Gombe State, Nigeria (2020) [38]. Other parts of Nigeria, beyond where *Anopheles stephensi* has been directly observed, have been deemed as climatically suitable for *Anopheles stephensi* based on Maxent random forest modeling using bioclimatic variables and elevation data [39] and via modeling people at risk for malaria transmission based on thermal transmission suitability. *Anopheles stephensi* thrives in urban environments, breeding in man-made water containers and exhibiting resistance to commonly used insecticides [40], which differs from the patterns of other malaria vectors [41]. *Anopheles stephensi* can survive in high temperatures and during the dry season [42]. The establishment of this species as a new vector throughout Africa has the potential to shift the epidemiology by exacerbating urban *Plasmodium* spp. transmission and undermining existing control measures.

One major strength of this study was the use of Thermo Fisher TAC assay technology, which provided a powerful screening tool in identifying infectious aetiologies that might otherwise have been missed by clinical diagnosis alone. This study also had some limitations. The Thermo Fisher TAC assays are proprietary. Neither the primers and probes used nor the genetic sequences used to design them are shared with the end users. Accordingly, as discrepancies between TAC results and hospital diagnostic procedures, or diagnoses occurred, the source of truth could not be ascertained, based on the available data. Further, TAC is a research-use only tool and cannot be used as a diagnostic tool in clinical settings. For these reasons, follow-up confirmatory testing is needed to confirm results presented here. Finally, in regions endemic for *Plasmodium* spp. including many parts of Nigeria, frequent exposure to the parasite is expected. This may or may not be the cause of the acute fever in the selected population. The acute fever could have been caused by one of the other pathogens detected (Table 4) or by other pathogens not included in SAFIAN. Determination of the actual cause of fever is beyond the scope of this study.

In conclusion, our study highlights regional and seasonal variability in *Plasmodium* spp. detection among febrile patients in Nigeria, with older children and individuals from crowded households at higher risk. A substantial burden of coinfections was observed, underscoring the value of multi-pathogen testing for febrile patients with nonspecific symptoms. While climate patterns aligned with seasonal trends, more granular data and longitudinal studies are needed. These findings support strengthening integrated febrile illness surveillance and adapting malaria control efforts to reflect changing epidemiologic and entomologic dynamics in Nigeria.

## Supplementary Material

ciaf468_Supplementary_Data

## Supplementary Data

Supplementary materials are available at *Clinical Infectious Diseases* online. Consisting of data provided by the authors to benefit the reader, the posted materials are not copyedited and are the sole responsibility of the authors, so questions or comments should be addressed to the corresponding author.
