# Supplementary material for: Epidemiology of Plasmodium spp. Detection Among Acute Febrile Illness Patients in Two Regions of Nigeria
Source: Clin Infect Dis. 2025 Nov 20;81(Suppl 4):S168–76. doi: 10.1093/cid/ciaf468 (PMC12631765; doi:10.1093/cid/ciaf468)
Supplement: ciaf468_Supplementary_Data [file ciaf468_supplementary_data.zip › Plasmodium Epi_Quiner_Supplemental Material 1_Final.docx]

Supplemental Table 1.

Results of the generalized linear mixed model, assessing the relationship between climate variables, with time lags, and percent positivity for Plasmodium spp.

| **Lag Time** | | **Effect** | | **Estimate** | | **Odds Ratio** | | **P-value** | | **Standard Error** | | **Degrees of Freedom** | | **t-statistic** |
| --- | --- | --- | --- | --- | --- | --- | --- | --- | --- | --- | --- | --- | --- | --- |
| 0 weeks | Intercept | | 2.9636 | | n/a | | 0.4845 | | 4.2212 | | 85 | | 0.70 | |
|  | Min Temp | | -0.01853 | | 0.98164 | | 0.8868 | | 0.1298 | | 85 | | -0.14 | |
|  | Max Temp | | -0.1018 | | 0.90321 | | 0.3342 | | 0.1048 | | 85 | | -0.97 | |
|  | Precipitation | | 0.04185 | | 1.04274 | | 0.5488 | | 0.06951 | | 85 | | 0.60 | |
|  | ISTH | | -0.5170 | | 0.59632 | | 0.3445 | | 0.5439 | | 85 | | -0.95 | |
|  | UATH | | 0 | | REF | | - | | - | | - | | - | |
| 1 week | Intercept | | 3.2064 | | n/a | | 0.4459 | | 4.1863 | | 84 | | 0.77 | |
|  | Min Temp | | -0.01447 | | 0.98563 | | 0.9136 | | 0.1330 | | 84 | | -0.11 | |
|  | Max Temp | | -0.1128 | | 0.89329 | | 0.2787 | | 0.1035 | | 84 | | -1.09 | |
|  | Precipitation | | 0.04943 | | 1.05068 | | 0.4549 | | 0.06585 | | 84 | | 0.75 | |
|  | ISTH | | -0.5622 | | 0.56995 | | 0.3071 | | 0.5472 | | 84 | | -1.03 | |
|  | UATH | | 0 | | REF | | - | | - | | - | | - | |
| 2 weeks | Intercept | | 3.5103 | | n/a | | 0.4180 | | 4.3122 | | 83 | | 0.81 | |
|  | Min Temp | | 0.03571 | | 1.03635 | | 0.7897 | | 0.1335 | | 83 | | 0.27 | |
|  | Max Temp | | -0.1501 | | 0.86065 | | 0.1570 | | 0.1051 | | 83 | | -1.43 | |
|  | Precipitation | | -0.01070 | | 0.98936 | | 0.8715 | | 0.06595 | | 83 | | -0.16 | |
|  | ISTH | | -0.5831 | | 0.55815 | | 0.2867 | | 0.5438 | | 83 | | -1.07 | |
|  | UATH | | 0 | | REF | | - | | - | | - | | - | |
| 3 weeks | Intercept | | 3.5037 | | n/a | | 0.4405 | | 4.5201 | | 81 | | 0.78 | |
|  | Min Temp | | 0.04702 | | 1.04815 | | 0.7383 | | 0.1403 | | 81 | | 0.34 | |
|  | Max Temp | | -0.1616 | | 0.85078 | | 0.1498 | | 0.1111 | | 81 | | -1.45 | |
|  | Precipitation | | 0.007606 | | 1.00764 | | 0.9101 | | 0.06713 | | 81 | | 0.11 | |
|  | ISTH | | -0.5671 | | 0.56718 | | 0.3107 | | 0.5559 | | 81 | | -1.02 | |
|  | UATH | | 0 | | REF | | - | | - | | - | | - | |
| 4 weeks | Intercept | | 1.3584 | | n/a | | 0.7716 | | 4.6640 | | 79 | | 0.29 | |
|  | Min Temp | | 0.09099 | | 1.09526 | | 0.5370 | | 0.1468 | | 79 | | 0.62 | |
|  | Max Temp | | -0.1322 | | 0.87616 | | 0.2601 | | 0.1165 | | 79 | | -1.13 | |
|  | Precipitation | | 0.04961 | | 1.05087 | | 0.4988 | | 0.07301 | | 79 | | 0.68 | |
|  | ISTH | | -0.6446 | | 0.52487 | | 0.2601 | | 0.5683 | | 79 | | -1.13 | |
|  | UATH | | 0 | | REF | | - | | - | | - | | - | |
| 5 weeks | Intercept | | 2.0932 | | n/a | | 0.6617 | | 4.7647 | | 77 | | 0.44 | |
|  | Min Temp | | 0.08686 | | 1.09075 | | 0.5508 | | 0.1450 | | 77 | | 0.60 | |
|  | Max Temp | | -0.1475 | | 0.86287 | | 0.2235 | | 0.1202 | | 77 | | -1.23 | |
|  | Precipitation | | 0.009193 | | 1.00924 | | 0.9008 | | 0.07353 | | 77 | | 0.13 | |
|  | ISTH | | -0.5590 | | 0.57179 | | 0.3351 | | 0.5763 | | 77 | | -0.97 | |
|  | UATH | | 0 | | REF | | - | | - | | - | | - | |
| 6 weeks | Intercept | | 1.0509 | | n/a | | 0.8423 | | 5.2636 | | 75 | | 0.20 | |
|  | Min Temp | | 0.05470 | | 1.05622 | | 0.6957 | | 0.1393 | | 75 | | 0.39 | |
|  | Max Temp | | -0.1018 | | 0.90317 | | 0.4636 | | 0.1382 | | 75 | | -0.74 | |
|  | Precipitation | | 0.05795 | | 1.05966 | | 0.4852 | | 0.08261 | | 75 | | 0.70 | |
|  | ISTH | | -0.4290 | | 0.65119 | | 0.4688 | | 0.5891 | | 75 | | -0.73 | |
|  | UATH | | 0 | | REF | | - | | - | | - | | - | |

ISTH = Irrua Specialist Teaching Hospital; UATH = University of Abuja Teaching Hospital.

n/a = not applicable; REF =reference
