# Supplementary figures and images for: Epidemiology of Plasmodium spp. Detection Among Acute Febrile Illness Patients in Two Regions of Nigeria
Source: Clin Infect Dis. 2025 Nov 20;81(Suppl 4):S168–76. doi: 10.1093/cid/ciaf468 (PMC12631765; doi:10.1093/cid/ciaf468)

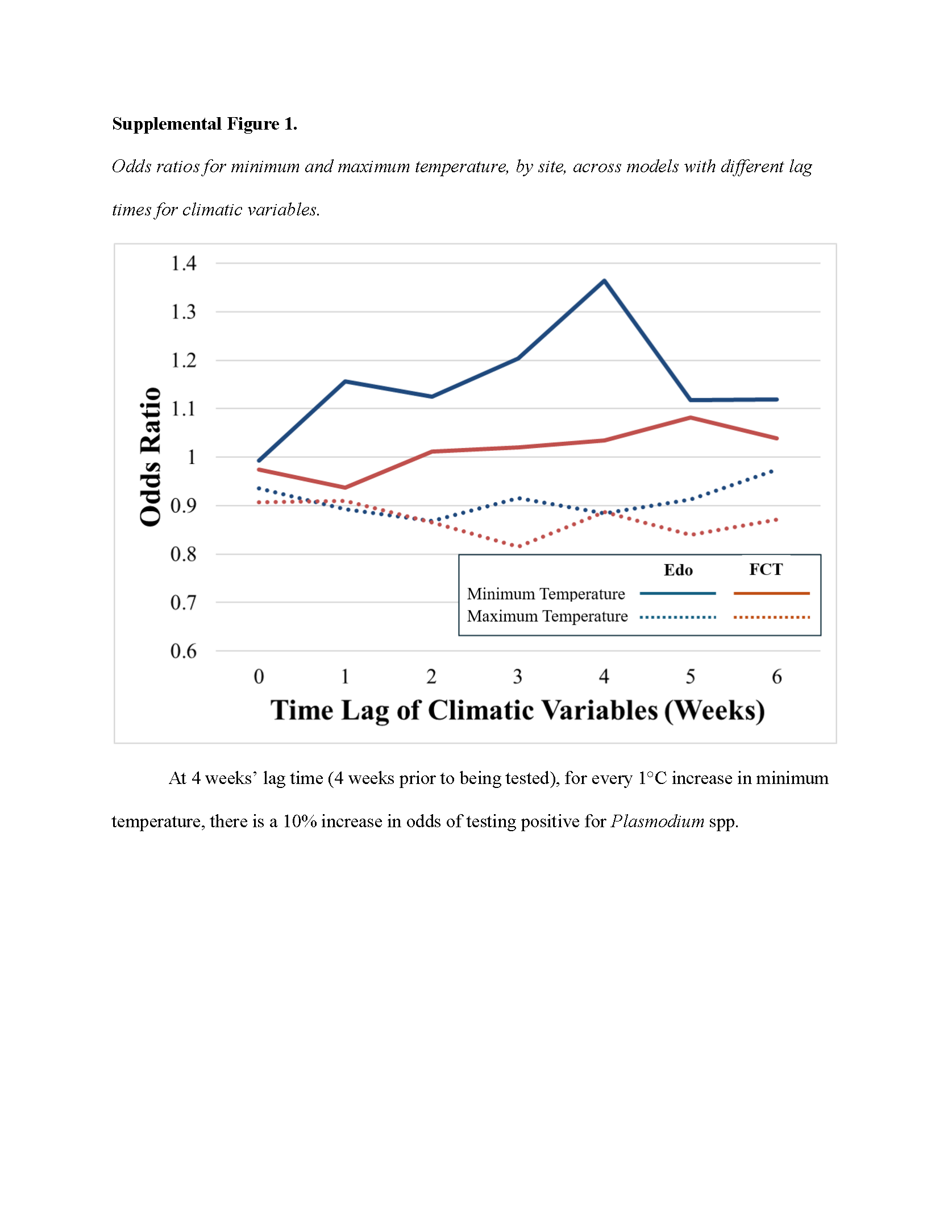

Supplement: ciaf468_Supplementary_Data [file ciaf468_supplementary_data.zip › Plasmodium Epi_Quiner_Supplemental Material 2_Final.tif]
